# Numerical Study on the Unstable Flow Dynamics of Wormlike Micellar Solutions past a Sphere in the Creeping Flow Regime

**DOI:** 10.3390/polym15102351

**Published:** 2023-05-17

**Authors:** Xiaohui Su, Zhiguo Wang, Jialun Liu, Song Wu

**Affiliations:** 1 School of New Energy, Xi’an Shiyou University, Xi’an 710065, China; zhgwang@xsyu.edu.cn (Z.W.); 181207@xsyu.edu.cn (J.L.); 2 School of Mechanical Engineering, Xi’an Shiyou University, Xi’an 710065, China; songwu@xsyu.edu.cn

**Keywords:** sphere, wormlike micellar solutions, viscoelasticity, flow instability

## Abstract

The flow dynamics of wormlike micellar solutions around a sphere is a fundamental problem in particle-laden complex fluids but is still understood insufficiently. In this study, the flows of the wormlike micellar solution past a sphere in the creeping flow regime are investigated numerically with the two species, micelles scission/reforming, Vasquez–Cook–McKinley (VCM) and the single-species Giesekus constitutive equations. The two constitutive models both exhibit the shear thinning and the extension hardening rheological properties. There exists a region with a high velocity that exceeds the main stream velocity in the wake of the sphere, forming a stretched wake with a large velocity gradient, when the fluids flow past a sphere at very low Reynolds numbers. We found a quasi-periodic fluctuation of the velocity with the time in the wake of the sphere using the Giesekus model, which shows a qualitative similarity with the results found in present and previous numerical simulations with the VCM model. The results indicate that it is the elasticity of the fluid that causes the flow instability at low Reynolds numbers, and the increase in the elasticity enhances the chaos of the velocity fluctuation. This elastic-induced instability might be the reason for the oscillating falling behaviors of a sphere in wormlike micellar solutions in prior experiments.

## 1. Introduction

The flows of particle-laden wormlike micellar solutions are widely encountered in industrial processes such as oil recovery, well drilling, personal care products and microfluidics [1,2,3,4,5,6,7,8]. The wormlike micelles in these kinds of solutions are large and flexible aggregates formed through spontaneous self-assembly by the amphiphilic surfactant molecules and salts dissolved in the water above a critical concentration [9]. The rheological properties of wormlike micellar solutions are more complex than those of polymer solutions or melts due to their viscoelasticity and the dynamic breakage and reformation of micelle chains, known as a “living polymer”. A systematic understanding of rheological properties of wormlike micellar solutions in various steady and transient flows is of fundamental interest and is the primary challenge of the research field. Many researchers have been focused on the rheology and flow patterns of these fluids, which exhibit distinct behaviors compared with Newtonian and the generalized Newtonian fluids [4,5,10,11,12,13,14,15]. When particles are dispersed in wormlike micellar solutions, the problem becomes more challenging.

The particle dynamics in wormlike micellar solutions have attracted a lot of attention from researchers. The settling of a single particle in various wormlike micellar solutions has been intensively investigated experimentally. A spherical particle was found to exhibit a continuously oscillating motion in the falling direction in wormlike micellar solutions, which never reaches a constant terminal velocity [5,16,17,18,19,20,21]. However, the reason for this phenomenon is thus far not very clear. Jayaraman and Belmonte [16] think that this unsteady motion of the sphere is caused by the formation and breaking of flow-induced structures, and some other researchers [17,18,21] think that the unsteady settling of the particle is associated with the breakage dynamics of the micelle network structure in the extensional wake of the particle.

Numerical simulations of particle dynamics, in which the viscoelasticity of these suspending fluids is considered, have been performed. Various viscoelastic constitutive equations, such as the UCM, Oldroyd-B, FENE and Giesekus equations, are generally used for the description of these viscoelastic fluids. These equations originate from the Maxwell’s spring and dashpot model with which we could effectively predict some typical viscoelastic phenomena of these fluids. The wormlike micellar chains, which are different from the micelles in the polymer solutions, undergo continuous scission and reformation under shear and extensional deformation, which in turn influences the rheology of the solutions and the flow dynamics. A few researchers concerned about the micelles’ scission and reformation physics, as well as the corresponding constitutive equations, are proposed. Vasquez et al. [22] built a network model (known as the “VCM” model) that is concerned with the scission and reforming of the micelle chains based on the Cates’ “living polymer” theory. They considered two active species of the elastic Hookean springs. The long chains can rupture into two short chains that can recombine into one long chain again. The rupture rate is dependent on the deformation rate of the fluid. The flows with the VCM constitutive model under the steady and transient, sheared and extensional deformations have been well studied by researchers [22,23,24].

Many of the studies have concentrated on the particle’s migration and chaining behaviors in microfluidics [6,7,25,26,27,28,29]. Pan and Glowinski [30] studied the spheres’ settling in Oldroyd-B fluids. They considered the setting of one, two and three spheres, and the chaining of the spheres was clarified. The settling velocity of a single sphere at a large ratio of the sphere diameter to that of the cylindrical vessel oscillates at the beginning, and then, it gradually reaches a steady terminal velocity. No continuous velocity oscillation is observed. Fernandes et al. [31] also found the initial instability of the settling velocity of a sphere with the Giesekus and FENE-CR constitutive model. Su et al. [32] investigated a sphere’s settling in Giesekus and FENE-P fluids and found that shear thinning weakens the oscillation of the settling velocity of the sphere compared with that in the Oldroyd-B fluids under identical rheological parameters. Fernandes et al. [33] developed a fluid–particle interaction force model through a direct simulation method by considering the volume fraction of the dispersed solid phase in Oldroyd-B fluids, which is applicable to the dense particle-laden flows in viscoelastic fluids. Qin et al. [34] recently developed a fully resolved simulation method based on the immersed boundary–lattice Boltzmann method using the Oldroyd-B and the Giesekus constitutive equations to investigate the particle dynamics in viscoelastic fluids, which shows good prospects for the simulation of flow behaviors of the particle-laden viscoelastic fluids. Sasmal’s research group [11,12,35,36] investigated the flow dynamics of the wormlike micellar solutions employing the two-species VCM model, and elastic instability was observed at very low Reynolds numbers, when the wormlike micellar solutions flowed around a spherical particle and cylinders. They found that the flow presents steady to periodic to quasi-periodic transitions when the shear Weissenberg number increases and that the elongation Weissenberg number also influences the flow pattern significantly. They thought that the unsteady motion downstream of the sphere was caused by the sudden rupture of long and stretched micelles in this region, because no unsteady flow is observed when the Giesekus models is used under otherwise identical conditions.

However, the main reason for the unstable flow of the wormlike micellar solutions past a sphere in the creeping flow regime is still unclear. Here, we use the classical Giesekus and the two-species VCM constitutive equations with the measured rheological parameters from the rheological experiments to explore the flow physics in order to reveal the essential flow dynamics, especially the role of elasticity in the complex flow phenomena of wormlike micellar solutions around a sphere.

This article is organized as follows. In Section 2, the mathematical models and numerical methodologies for viscoelastic fluids flowing around a sphere are presented. In Section 3, the numerical methods and procedures are validated against the well-documented theories and published data, and the flow dynamics are presented and discussed. Finally, some concluding remarks are given in Section 4.

## 2. Mathematical Model and Numerical Considerations

### 2.1. Mathematical Model

The governing equations for the transient, incompressible, and isothermal flow of wormlike micellar solutions include the continuity equation, the momentum equation, and the constitutive equations that define the extra elastic stress contribution, as reported by previous research [7,22,30,33,37].

Continuity equation,

(1) ∇⋅u=0

Momentum equation,

(2) $$\rho (\frac{\partial u}{\partial t}+u\cdot \nabla u)=\nabla (-pI+\mu_{s}(\nabla u+{\left(\nabla u\right)}^{T})+\tau )$$

where *ρ* is the fluid density, ***u*** is the velocity vector, *t* is the time, *μ*_s_ is the solvent viscosity, and ***τ*** is the extra viscoelastic stress, which is derived from the constitutive equation.

The two-species VCM model, which was formulated by Vasquez, McKinley and Cook [22], is employed here to model the extra viscoelastic stress by taking into account the chains’ breakage and recombination physics. The details about the VCM constitutive equations are given in References [22,23,24]. As they reported, the extra viscoelastic stress is calculated by introducing the number density and the conformation tensor of the micelles. It is as follows:

(3) $$\tau =G_{0}[(A+2B)-(n_{A}+n_{B})I]$$

where *G*_0_ is the elastic modulus of the micelle, *n*_A_ and ***A*** are the number density and the conformation tensor of the long micelles, respectively, and *n*_B_ and ***B*** are the number density and the conformation tensor of the short micelle, respectively.

The number density transport equations of the long chains and short chains are as follows:

(4) $$\frac{\partial n_{A}}{\partial t}+u\cdot \nabla n_{A}=2D_{A}\nabla^{2}n_{A}+\frac{1}{2\lambda_{A}}c_{B}n_{B}^{2}-\frac{c_{A}n_{A}}{\lambda_{A}}$$

(5) $$\frac{\partial n_{B}}{\partial t}+u\cdot \nabla n_{B}=2D_{B}\nabla^{2}n_{B}-\frac{c_{B}n_{B}^{2}}{\lambda_{A}}+2\frac{c_{A}n_{A}}{\lambda_{A}}$$

where *D_A_* and *D_B_* are the diffusion coefficients of the long chains and the short chains, respectively, *λ_A_* is the relaxation time of the long chains, and *c_A_* and *c_B_* are the breakage rate of the long chains and the recombination rate of the short chains, respectively.

The breakage rate is related to the nonlinear factor of the chain breakage and the local strain rate:

(6) $$c_{A}=c_{A_{Eq}}+\frac{\xi \mu }{3}\left(\dot{\gamma }:\frac{A}{n_{A}}\right)$$

where $c_{A_{Eq}}$ is the breakage rate of the long chains under the equilibrium state. *μ* = 1 + $c_{A_{Eq}}$, $\dot{\gamma }$ is the strain rate tensor, and *ξ* is a physical quantity that characterizes the nonlinear effect of the breakage of the long chains. The recombination rate of two short chains into a long chain is assumed to be equal to the equilibrium rate $c_{B}=c_{B_{Eq}}$.

The conformation tensors of species ***A*** and ***B*** are determined as

(7) $$\lambda_{A}\overset{\nabla }{A}+A-n_{A}I-\lambda_{A}D_{A}\nabla^{2}A=c_{B}n_{B}B-c_{A}A$$

(8) $$\varepsilon \lambda_{A}\overset{\nabla }{B}+B-\frac{n_{B}}{2}I-\varepsilon \lambda_{A}D_{B}\nabla^{2}B=-2\varepsilon c_{B}n_{B}B+2\varepsilon c_{A}A$$

where *ε* = *λ_B_*/*λ_A_*, *λ_B_* is the relaxation time of the short chains.

The effective relaxation time of the VCM fluid is

(9) $$\lambda =\frac{\lambda_{A}}{1+C_{A_{Eq}}}$$

The single-species Giesekus constitutive equation is as follows:

(10) $$\tau +\overset{\nabla }{\tau }+\alpha \frac{\lambda }{\mu_{p}}(\tau \cdot \tau )=\mu_{p}(\nabla u+\nabla u^{T})$$

where *α* is the mobility factor, *λ* is the relaxation time, and *μ*_p_ is the polymer viscosity.

The dimensionless quantities, Reynolds number *Re*, and Weissenberg number *Wi* are given as

(11) $$Re=\frac{\rho dU_{\mathrm{in}}}{\mu_{0}}$$

(12) $$Wi=\frac{\lambda U_{\mathrm{in}}}{d}$$

where *U*_in_ is the velocity of the inlet main stream.

The drag coefficient of a sphere in viscoelastic fluids is defined by integrating the total stress on the sphere surface as

(13) $$C_{D}=\frac{2}{\rho U_{\mathrm{in}}^{2}A}\int_{S}\left(-pI+\mu_{S}(\nabla u+{\left(\nabla u\right)}^{T})+\tau \right)\cdot n\cdot e_{z}dS$$

where *A* is the projected area of the sphere along the flow direction, ***I*** is the unit identity tensor, ***n*** is the unit normal vector of the sphere surface S, and ***e***_*z*_ is the unit vector along the flow direction.

### 2.2. Numerical Methods

**Figure 1:**
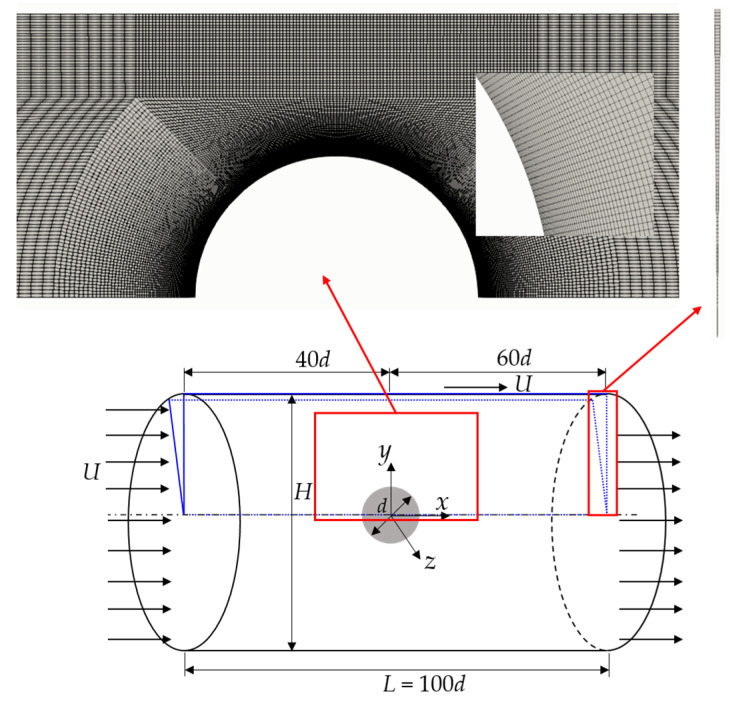
Schematic of the current problem and the typical mesh used in the simulations.

We aim to investigate the flow characteristics of the fluids past a sphere in the creeping flow regime. Considering the axial symmetry of the whole computational domain, a wedgy geometry, with which the mesh is created, is extracted from the actual flow domain, as shown in Figure 1. The main stream with perpendicular velocity *U*_in_ to the inflow face flows into a circular tube of diameter *H* with a sphere of diameter *d* fixed at the center of the tube. The lengths of the inlet region and the outlet region of the tube are 40*d* and 60*d*, respectively, ensuring that the flows are fully developed and that the stresses are relaxed sufficiently. A structural grid is used, and the regions with large gradients of velocity and stress are refined, as shown in Figure 1.

The OpenFOAM toolbox based on the finite volume method and a recently developed solver rheoTool-4.1 are used to solve the governing equations. The diffusion and gradient terms are all discretized with the central differencing scheme. The convective terms are discretized with the CUBISTA (Convergent and Universally Bounded Interpolation Scheme for Treatment of Advection) scheme to improve the convergence of the numerical solutions. The SIMPLE method is employed for velocity–pressure coupling. The error tolerance is determined below 10^−8^.

The procedure to solve the differential equations is summarized in the following steps:

1. For given initial fields of velocity ***u***, pressure *p* and stress ***τ***, the explicit calculations of the pressure gradient and the stress divergence are carried out, and subsequently, the momentum equation is solved implicitly for each component of the velocity vector, computing a new velocity field estimate u*.
2. With the new velocity values u*, the new pressure field p* is estimated, and subsequently, the correction of the velocity is carried out, leading to a new velocity field u**, which satisfies the continuity equation. The SIMPLE algorithm is used to obtain p* and u**.
3. With the corrected velocity field u**, the new estimate τ* for the stress tensor field is calculated by solving the specified constitutive equation.
4. Steps 1, 2 and 3 are repeated recursively within each time step in order to generate more accurate solutions in transient flow. For this, ***u***, *p* and ***τ*** are updated with u**, p* and τ*, respectively.

No slip condition for velocity, ***u*** = ***u*** (0, 0, 0), is applied at the sphere surface. The fixed velocity, ***u*** = ***u*** (*U*_in_, 0, 0), is used at the tube inlet and the tube wall. At the tube outlet, *p* = *p*_out_ and zero-velocity gradient are imposed. For the VCM model, no flux boundary, ***n***∙∇*ϕ* = 0, is used for the number density and the confirmation tensor components of the micelles at the sphere surface, tube wall and the tube outlet. At the tube inlet, the number densities of the micelles are *n*_A_ = 1, $n_{B}=\sqrt{2C_{A_{Eq}}/C_{B_{Eq}}}$, and the conformation tensors are ***A*** = (1 0 0 1 0 1), ***B*** = (*n_B_*/2 0 0 *n_B_*/2 0 *n_B_*/2). For the Giesekus model, the linear extrapolation method is used for the extra viscoelastic stress components at the sphere surface and the channel wall. The zero extra viscoelastic stress components are used at the tube inlet. Zero gradient condition is applied at the tube outlet. The time step is fixed at 5 × 10^−5^ s, ensuring that the courant number is below 0.1 and helping to improve convergence and accuracy.

## 3. Results and Discussion

### 3.1. Mesh Independence Study

The solution independence on the grids is first examined. Considering the important influence of the dominant elastic stress on the flow in the wormlike micellar solutions, the extensional elastic stress along the *x*-axis in the wake of the sphere and the calculated drag forces on the sphere are chosen as the desired quantities for analysis. The grid convergence index (*GCI*) proposed by Roache [38,39] is recommended to evaluate the numerical accuracy resulting from the mesh resolution and has been used in some previous research [40,41]. *GCI* evaluation of the chosen quantities for three grids with different cell densities is performed.

In the *GCI* methodology, the refinement ratio of the given grids is defined as

(14) $$r={\left(N_{f+1}/N_{f}\right)}^{1/D}$$

where *N_f_*_+1_ is the cell quantity of the grid after refinement, *N_f_* is the cell quantity before refinement, and *D* is equal to two, considering that the grid is created in an-axisymmetric two-dimensional plane. Then, the grid convergence index for the chosen quantities can be derived using the following equation

(15) $$GCI=\frac{F_{S}\left|e\right|}{r^{p}-1}$$

where *F_s_* is a safety factor, and it is suggested as 1.25 [39], because three grids are used here. |*e*| is the absolute value of the relative error between the solutions obtained with the two grids. The parameter *p* is the order of the convergence, and it is evaluated using an iterative method [39], because the refinement ratio *r* is not a constant here. Specifically, the iterative equations are as follows

(16) $$\beta =\frac{\left(r_{12}^{p_{\mathrm{li}}}-1\right)}{\left(r_{23}^{p_{\mathrm{li}}}-1\right)}\frac{e_{23}}{e_{12}}$$

(17) $$p=\omega p_{\mathrm{li}}+(1-\omega )\frac{\ln\beta }{\ln r_{12}}$$

where *p*_li_ is the value of *p* in the latest iteration, *ω* is the relaxation factor determined as 0.5, and *r*_12_ and *r*_23_ are the refinement ratio of the fine grid (#1) to the base grid (#2) and the base grid (#2) to the coarse grid (#3), respectively.

Results of the *GCI* analysis for the two quantities are shown in Table 1. For extensional elastic stress *τ_xx_*, *GCI*_23_ /(*r^p^GCI*_12_) is obtained as 0.99998, indicating that the solutions are well within the asymptotic range of convergence. Furthermore, the analysis for the *GCI* values of the drag coefficient (with relatively smaller values) present a similar conclusion.

### 3.2. Model Validation

Firstly, the dimensionless quantities such as the Reynolds number and the Weissenberg number in existing simulations were chosen more or less arbitrarily to perform the numerical simulations to ensure the stability of the numerical simulation. The tests show that the tiny variations in the two quantities do not change the simulation results significantly. Therefore, the numerical schemes for the simulations are stable and robust.

**Figure 2:**
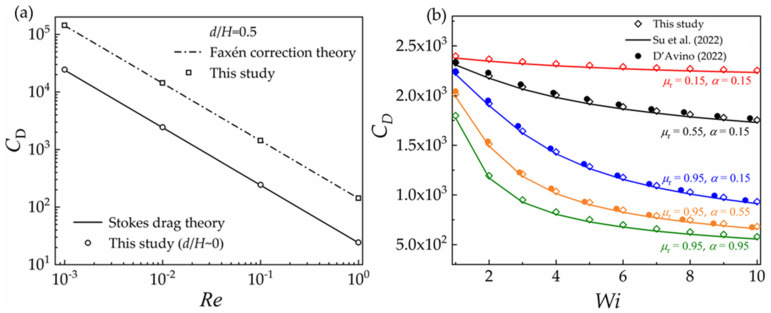
Comparison of the drag coefficients of theory predictions and the literature data [37,42] with the current numerical data: (**a**) VCM model (*G*_0_ = 0); (**b**) Giesekus model (*Re* = 0.01).

Considering the lack of the generally recognized simulation data for the VCM model, we performed simulations using the VCM constitutive equation in which a vanishing elastic module, *G*_0_, is assumed. Then, the constitutive equation degrades to the Newtonian type. As we know, the drag forces of the Newtonian fluids on a sphere at low Reynolds numbers obey the Stokes theory. Therefore, the drag coefficients calculated from the simulated results by assuming a vanishing elastic module, *G*_0_, in the VCM constitutive equation are compared with the predictions of Stokes theory and Faxén correction theory to validate the above numerical methods and procedures. The simulation results are compared with the theory predictions, as shown in Figure 2a, where the simulation data agree well with the theory predictions.

The simulation data with the Giesekus constitutive model are also validated against the results in previous studies [37,42], as shown in Figure 2b. *μ*_r_ denotes the ratio of the polymer viscosity to the zero shear viscosity. It shows good agreement between the current simulation results and the literature data. Thus, the current numerical methods are qualified for the simulations.

### 3.3. The Rheological Properties of the Constitutive Equations

The rheological parameters for the two-species VCM constitutive equation are from rheological experiments by Zhou et al. [24]. The non-dimensional parameters are *β* = *μ*_S_/*μ*_0_ = 2.2 × 10^−5^, *μ* = 1.8, *ε* = 7 × 10^−5^, *ξ* = 0.1, $n_{B}^{0}$ = 1.13, *δ* = 0.001. The solvent viscosity *μ*_S_ is 1 × 10^−3^ Pa·s. The material parameters (*G*_0_ = 27.2 Pa, *λ* = 1.67 s) from the experimental measurements are used. The values of the zero shear viscosity, the polymer viscosity ratio and the relaxation time for the single Giesekus model are identical to those of the two-species VCM model. The rheological curves for these parameters are obtained by calculating the stress–strain rate using the rheoTestFoam solver in rheoTool.

**Figure 3:**
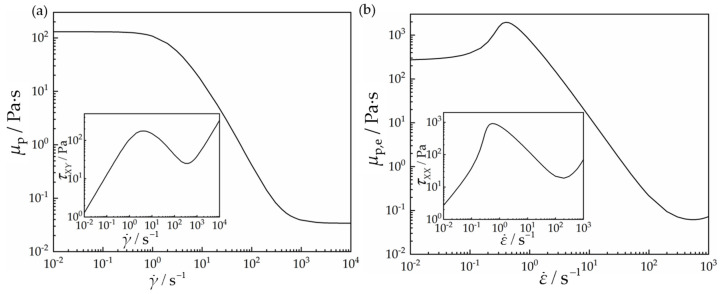
The rheological properties of the VCM model under (**a**) planar homogeneous shear deformation and (**b**) planar uniaxial extensional deformation.

Figure 3a presents the variation in shear viscosity and stress with the shear strain rate. It shows that the shear stress almost increases linearly with the shear strain rate, when the shear strain rate is less than 1 s^−1^. It reaches a local maximum and then declines gradually with the increase in the shear strain rate, and it almost increases linearly once again, when the shear strain rate is more than 10^3^ s^−1^. The shear stress shows a strong nonlinear variation with the shear strain rate in the range 1–10^3^ s^−1^. It is clearly seen from Figure 3a that the shear viscosity is a constant when the shear strain rate is less than 1 s^−1^ and is more than 10^3^ s^−1^, and shear thinning occurs when the shear strain rate is in the range 1–10^3^ s^−1^.

The extensional rheology is also obtained for given extensional strain rates. Figure 3b shows the variation in extensional viscosity and stress with the extensional strain rate. The variation in extensional viscosity with the extensional strain rate also presents strong nonlinearity. It increases rapidly from less than 300 Pa·s to 2000 Pa·s, when the extensional strain rate increases from 0.01 s^−1^ to 0.4 s^−1^, and then it declines, and it is below 0.1 Pa·s when the extensional strain rate is larger than 300 s^−1^. The extensional viscosity changes with four orders of magnitude within the range of the extensional strain rate investigated.

**Figure 4:**
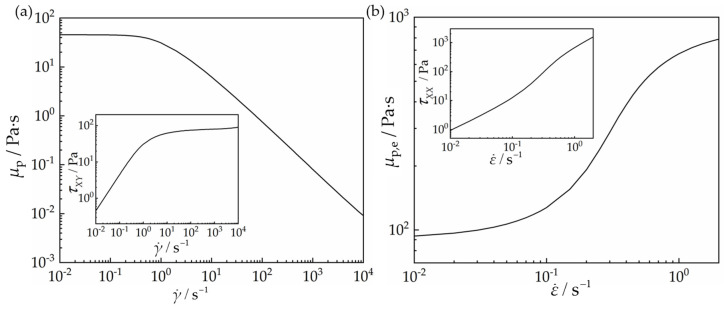
The rheological properties of the Giesekus model under (**a**) planar homogeneous shear deformation and (**b**) planar uniaxial extensional deformation.

The rheological properties of the Giesekus model are shown in Figure 4. It presents shear thinning as well as the VCM model. Extension hardening is also observed within the range of an extensional strain rate of 0.01–1 s^−1^. The numerical solution becomes divergent when the extensional strain rate is larger than 1 s^−1^. Despite the inaccessibility of the numerical solutions at higher extensional strain rates, it shows that the extensional viscosity has a significant increase, when the extensional strain rate changes from 0.01 s^−1^ to 1 s^−1^.

Therefore, the two-species VCM and the single Giesekus constitutive equations both exhibit strong shear thinning and extension hardening. However, the VCM model presents the extension hardening at first, followed by subsequent extension thinning. It shows more complex extensional rheological properties than those of the Giesekus model.

### 3.4. The Flow Dynamics around a Sphere

**Figure 5:**
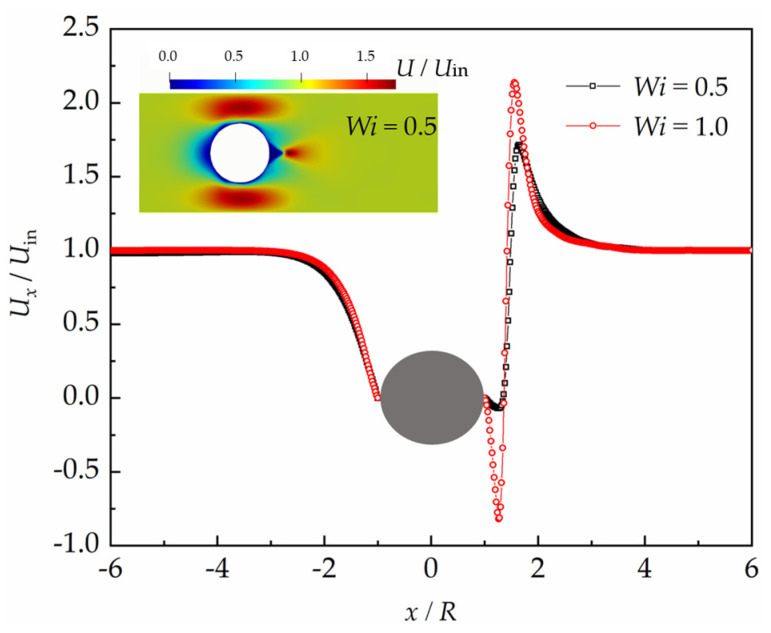
Contour plot of the calculated velocity (in the subfigure) and the non-dimensional velocity along the *x*-axis before and after the sphere with the VCM model.

The numerical simulations were performed with the Reynolds number within the range from 10^−6^ to 10^−2^. The flows are calculated with the ratio of the diameter of the sphere to that of the tube of 0.5. The typical flow pattern around the sphere is shown in Figure 5. It is significantly different from the flow fields of the Newtonian fluids at low Reynolds numbers. The velocity magnitude exhibits an obvious asymmetry between the upstream and downstream region of the sphere along the *y*-axis, which is distinct from symmetrical distribution of the velocity magnitude of Newtonian fluids. In addition to that, there exist a downstream region with a relatively large velocity and a velocity peak that exceeds the velocity magnitude of the main stream.

The asymmetrical velocity distribution can be seen clearly from the velocity along the *x*-axis before and after the sphere, as shown in the velocity contour in Figure 5. The velocity becomes smaller and smaller, when the fluid gradually approaches the front stagnation point of the sphere. The velocity after the sphere changes very significantly. A negative value of the velocity along the *x*-axis occurs, which means that the fluid flows in an opposite direction to the main stream in this region. The velocity magnitude increases with the distance apart from the sphere until it reaches a local maximum, and then, it decreases gradually. The flow changes its direction with the further increase in the distance apart from the sphere, and then, the velocity magnitude increases again. It increases rapidly up to a maximum, which exceeds the velocity of the main stream, and subsequently reduces once more. It declines eventually to a magnitude equal to the velocity magnitude of the main stream.

**Figure 6:**
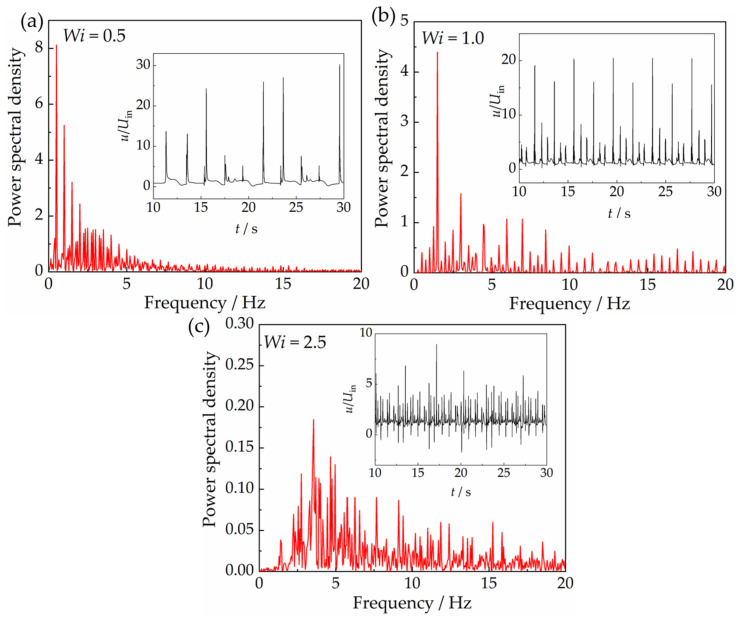
Fluctuations of the velocity in the *x* direction (in the subfigures) and the power spectral density at the coordinate point *X* (*x*/*d*) = 1, *Y* (*y*/*d*) = 0 with the VCM model for different Weissenberg numbers, (**a**) *Wi* = 0.5, (**b**) *Wi* = 1.0, (**c**) *Wi* = 2.5.

The variations in velocity in the *x* direction with time at the coordinate point (*X* (*x*/*d*) = 1, *Y* (*y*/*d*) = 0) are shown in Figure 6. The velocity here is non-dimensionalized by dividing the inlet velocity *U*_in_ with the calculated velocity *u* in the *x* direction. It shows the velocity fluctuates continuously with time, and it presents quasi-periodic regularity. The velocity fluctuation was found to occur when the Weissenberg number exceeds 0.2. The value of the critical Weissenberg number at which velocity fluctuation starts to occur here is smaller than that of Sasmal [11], mainly because of the larger parameter *ξ* used by them. *ξ* is a nonlinear parameter for characterizing the nonlinear response of the longer elastic chain. The chain scission rate increases with the increase in *ξ*, i.e., the longer chains break faster, which releases the elastic energy more easily; thus, elastic instability is less prone to occur.

The fluctuation signal of the velocity has one principal component, which has the largest amplitude and a relatively low frequency. There are also several significant components with relatively large amplitude and small frequency. Besides the several main components, there exists a large amount of minor components with very small amplitude, which are mainly located in the high-frequency section, indicating that the velocity fluctuation is prone to being stochastic, especially when the Weissenberg number is higher. In addition, the fluctuation amplitude of the velocity decreases obviously when the Weissenberg number increases. This fluctuation in velocity in the wake of the sphere is analogous to the results obtained by Sasmal [11]. The velocity fluctuation in the wake is quasi-periodic, and it tends to become stochastic when elasticity increases.

**Figure 7:**
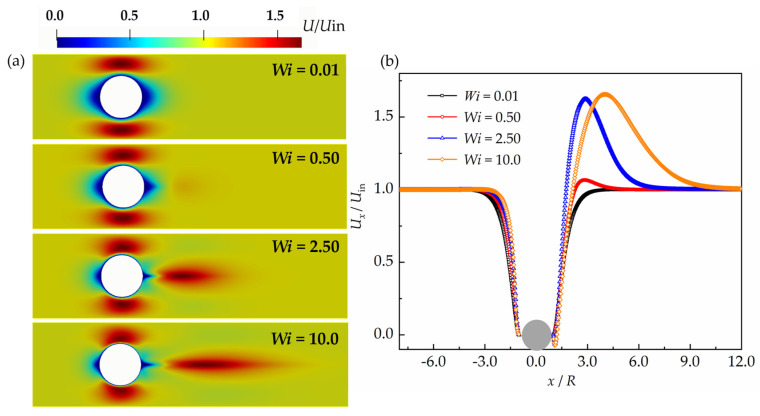
(**a**) Contour plot of calculated velocity and (**b**) non-dimensional velocity along the *x*-axis before and after the sphere with the Giesekus model.

The flows around a sphere with the Giesekus constitutive equation are also studied. The flow pattern around a sphere with the Giesekus model is shown in Figure 7a. It shows a similarity qualitatively with the results using the VCM model. When the Weissenberg number is 0.01, the flow is similar to the situation with Newtonian fluids. While the velocity in the wake of the sphere becomes very different when the Weissenberg number increases. There exists a stretched wake with a large velocity gradient and a region with high velocity that exceeds the main stream velocity in the wake of the sphere. The large velocity region in the wake is enlarged when the Weissenberg number increases. This can also be seen in Figure 7b. The gap between the maximum velocity magnitude and the main stream velocity increases significantly when the Weissenberg number increases from 0.5 to 2.5; then, it changes very little when the Weissenberg number increases from 2.5 to 10. It shows that the region with large velocity exceeding the main stream velocity is enlarged when the Weissenberg number increases, indicating a longer stretched wake formed after the sphere.

**Figure 8:**
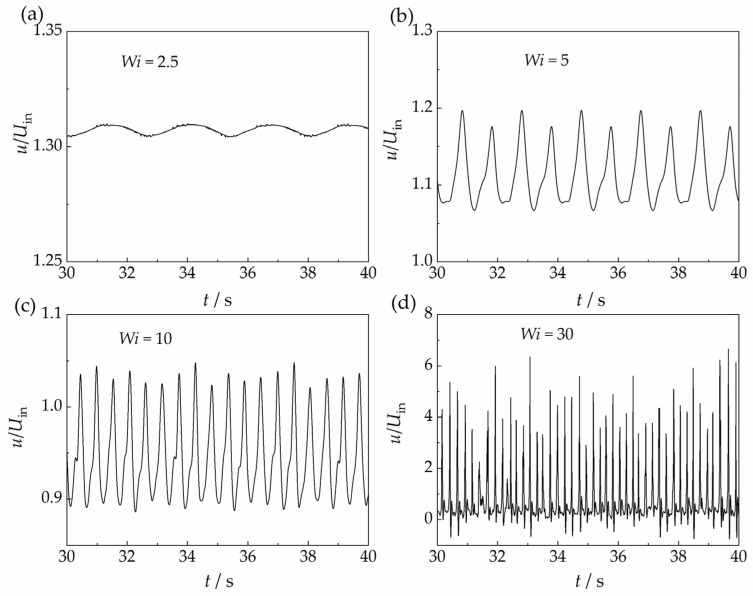
Fluctuations in velocity along the *x*-axis at the coordinate point *X* (*x*/*d*) = 1, *Y* (*y*/*d*) = 0 with the Giesekus model, (**a**) *Wi* = 2.5, (**b**) *Wi* = 5, (**c**) *Wi* = 10, (**d**) *Wi* = 30.

The velocity in the wake of the sphere also shows significant instability for the Giesekus model, as shown in Figure 8, which shows qualitative similarity to the results with the VCM model. The velocity magnitude changes continuously with time. The fluctuation amplitude is very small when the Weissenberg number is relatively small, and it increases when the Weissenberg number increases. The maximum amplitude of the velocity fluctuation reaches above 6.0, indicating that the velocity there is much larger than the main stream velocity. This causes the so-called “negative wake” for a sphere that translates steadily in the wormlike micellar solutions, as reported by Chen and Rothstein [21] in experiments and as predicted in simulations using the Giesekus model by Su et al. [37]. The velocity in the wake also fluctuates with time. After a pulse, the velocity decreases rapidly until it vanishes, and then, a velocity with a direction opposite to the direction of the main stream may appear, when the Weissenberg number is 30.

**Figure 9:**
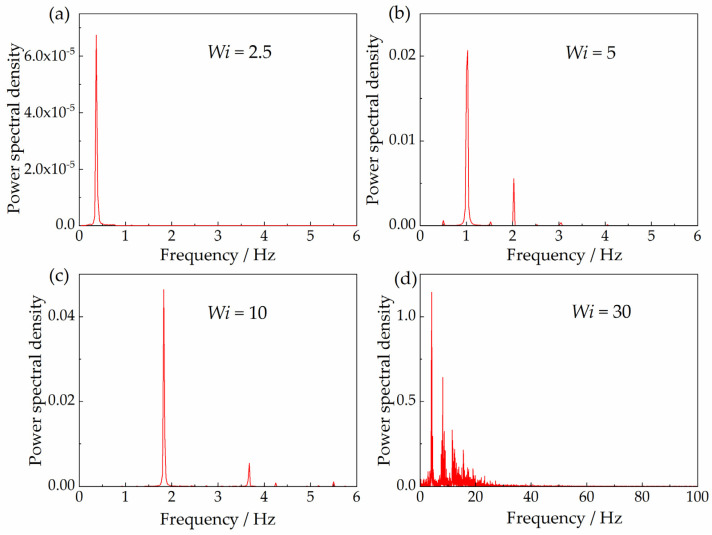
Power spectral density of the fluctuations in velocity along the *x*-axis at the coordinate point *X* (*x*/*d*) = 1, *Y* (*y*/*d*) = 0 with the Giesekus model, (**a**) *Wi* = 2.5, (**b**) *Wi* = 5, (**c**) *Wi* = 10, (**d**) *Wi* = 30.

It can be seen that the velocity fluctuation in the wake of the sphere is quasi-periodic from the power spectral density of the fluctuation signals, as shown in Figure 9. There is only one significant fluctuation component when the Weissenberg number is relatively small. However, the number of fluctuation components increases with the increase in the Weissenberg number. There is one principal component with the largest amplitude and several minor components with smaller amplitudes, indicating the quasi-periodic pattern of the velocity fluctuation. The number of minor fluctuation components increases significantly when the Weissenberg number increases up to 30, indicating that the fluctuation becomes more chaotic. Therefore, the flow instability is enhanced at stronger elasticity, and it is prone to transitioning into a complete random pattern when the elasticity is strong enough.

We inferred that this chaotic velocity fluctuation in the wake of the sphere is associated with the sphere’s oscillating settling behaviors, which were experimentally reported by researchers [5,17,18,20], because both oscillating behaviors exhibit analogous chaotic features qualitatively. The frequencies of the velocity oscillations for both the current simulations (see Figure 9) and experiments results (see Figure 3b in reference [18]) have one principal component with the largest amplitude and several minor components with smaller amplitudes. The number of minor components increases with the increase in elasticity, indicating a more chaotic oscillation. In addition, the values of both the frequency and amplitude of the principal component increase with the increase in elasticity.

The fluctuating velocity field in the wake of the sphere at low Reynolds numbers using the Giesekus constitutive model shows that the flow instability is caused by the elasticity of the wormlike micellar solutions. The breakage dynamics of the micelle chains could affect the variation in elastic stress and consequently affect the intensity of the flow instability.

## 4. Conclusions

In this study, the two species, micelles scission/reforming Vasquez–Cook–McKinley, and the single-species Giesekus constitutive equations, are used to investigate the flow dynamics of wormlike micellar solutions passing by a sphere in the creeping flow regime with numerical methods. The steady shear and extension rheology of the fluids are obtained, and the flow pattern and dynamics are analyzed and discussed.

The two constitutive models both exhibit shear thinning and extension hardening, while the VCM model presents extension hardening and subsequent extension thinning. The flow becomes very unstable above a critical Weissenberg number. When the fluids flow past a sphere at very low Reynolds numbers, there exists a region with higher velocity than the main stream in the wake of the sphere, which forms a stretched wake with a large velocity gradient. The length of the wake increases when the Weissenberg number increases. The physical quantities in the wake of the sphere are found to exhibit a quasi-periodic fluctuation not only for the two-species micelles scission/reforming VCM models, which was observed in previous numerical studies, but also for the single-species Giesekus model, with which the flow instability is observed in this study. The results indicate that it is the elasticity of the wormlike micellar solutions that induces the distinct flow pattern and the instability at low Reynolds numbers. In addition, the increase in elasticity enhances the chaos of the velocity fluctuation. These velocity fluctuations in the wake of the sphere show qualitative similarity with the fluctuations of the falling velocity of a sphere in wormlike micellar solutions that have been intensively observed in previous experiments. Elastic instability is the main reason for oscillating falling behaviors of a sphere in wormlike micellar solutions.

## Figures and Tables

**Table 1:** *GCI* evaluated for the extensional elastic stress ***τ***_xx_ at coordinate point (*x*/*d* = 0.8, *y*/*d* = 0) and the drag coefficient *C*_D_ of the sphere.

Index	Grids Adopted in Simulations	Extensional Elastic Stress *τ*_xx_ at Coordinate Point (*x*/*d* = 0.8, *y*/*d* = 0)/Pa	*GCI* *τ*_xx_	*C*_D_	*GCI* *_C_*_D_
#1	Fine	33.571	1.624%	1.58378 × 10^6^	0.001%
#2	Base	33.235	2.874%	1.58311 × 10^6^	0.051%
#3	Coarse	32.717	-	1.56193 × 10^6^	-

## Data Availability

Not applicable.

## Nomenclature

***u***	fluid velocity, m·s^−1^
*ρ*	fluid density, kg·m^−3^
*t*	time, s
*p*	pressure, Pa
***I***	unit identity tensor
***τ***	viscoelastic stress tensor, Pa
*μ*_S_	viscosity from solvent contribution for viscoelastic fluids, Pa·s
*G*_0_	elastic module of the micelles, Pa
*n*_*A*,*B*_	density number of long and short micellar chains
***A***, ***B***	conformation tensor of long and short micellar chains
*D*_A, B_	diffusion coefficient of long and short micellar chains, m^2^·s^−1^
*c*_*A*, *B*_	rupture and recombination rate of long and short micellar chains
*c*_*A*,*B*Eq_	equilibrium rupture rate of micellar chains
*λ*_*A*,*B*_	relaxation time of long and short micellar chains, s
*ε*	ratio of relaxation time of short to long micellar chains
*μ*	ratio of relaxation time of long micellar chains to effective relaxation time
$\dot{\gamma }$	strain rate, s^−1^
*ξ*	scission energy of long micelles
*μ*_p_	shear viscosity of polymer, Pa·s
*μ*_p,e_	extensional viscosity of polymer, Pa·s
*Re*	Reynolds number
*Wi*	Weissenberg number
*U*_in_	inlet velocity of fluid, m·s^−1^
*λ*	effective relaxation time, s
*d*	diameter of sphere, m
*H*	diameter of cylindrical channel, m
***n***	unit normal vector on the wall
*C*_D_	drag coefficient of the sphere
*A*	projected area of the sphere along the flow direction
*I*	unit identity tensor
***e***_*z*_	unit vector along the flow direction
*GCI*	grid convergence index
*r*	refinement ratio of the grid
*p*	order of the convergence of grid
*e*	relative error of quantities obtained from two grids
*μ*_r_	ratio of polymer viscosity to zero shear viscosity
*β*	ratio of solvent viscosity to zero shear viscosity
***τ***_*XX*,*XY*_	stress components, Pa
